# (Escaping) the paradox of scientific storytelling

**DOI:** 10.1371/journal.pbio.2006720

**Published:** 2018-10-09

**Authors:** Michael F. Dahlstrom, Dietram A. Scheufele

**Affiliations:** 1 Iowa State University, Ames, Iowa, United States of America; 2 University of Wisconsin–Madison, Madison, Wisconsin, United States of America; 3 Morgridge Institute for Research, Madison, Wisconsin, United States of America

## Abstract

Compelling stories about science can motivate people to engage and respond to relevant problems facing society. While science plays a unique role in society, providing the best available evidence for policy choices, understanding the world, and informing citizens’ daily lives, it does not hold any intrinsic advantage in creating captivating stories for mass audiences. Instead, science must compete with other storytellers, many of whom are not bound to scientific evidence. This presents a paradox—how can science preserve its credibility as curator of knowledge while engaging audiences with a communication format that is agnostic to truth?

This Perspective is part of the *Confronting Climate Change in the Age of Denial Collection*.

## Introduction

Storytelling is the creation and sharing of specific narrative messages. A narrative message is a distinct communication format structured around a character who experiences events over time, often overcoming conflict on the way. Because narrative messages portray larger phenomena through an individual’s experience, narrative messages are predisposed to depict information in ways audiences can identify with both cognitively and emotionally [1,2]. Strongly constructed narrative messages can therefore be highly persuasive [3]. A number of science-related organizations, both academic and not, are promoting narrative messages as influential tactics to engage audiences in support of science [4,5].

However, storytelling is also a way of thinking to evaluate information. Most direct experience is stored in memory as narrative cases, and we often interpret new information not on its own merit but by trying to integrate it with the existing narratives and mental schemas we already use to understand the world [6]. In this sense, a narrative way of thinking endows single cases and anecdotes with significant weight toward evaluating evidence. Not all information is processed through this narrative way of thinking, but research suggests that it is the default mode of human thought if not countered with intent [7]. In this sense, a narrative way of thinking is a distinctly unscientific way of knowledge production because it focuses on particular instances rather than considering the full range of possibilities. “[T]he plural of the word anecdote,” as Kernaghan and Kuruvilla [8] put it dryly, “is not data.” Because of this, scientists sometimes resist telling stories, feeling they are manipulative or are oversimplifications.

## The paradox of scientific storytelling

So when are narratives useful and appropriate tools for science communication? And when might the use of scientific storytelling be antithetical to the goals and mission of science? To unpack this paradox, we suggest comparing how these concepts of storytelling (narrative messages versus a narrative way of thinking) align with different underlying goals for communicating science with external audiences [9].

### Knowledge, attitudes, and behaviors

If the goal of communicating science is to transmit knowledge about scientific facts, then narrative messages can be an effective tactic to a larger end. Scientists could use narrative messages to engage and motivate audiences to learn about climate change or vaccine safety, for example. Likewise, if the goal of communicating is to influence attitudes or behaviors toward science, scientists could use narrative messages to demonstrate and encourage certain attitudes or behaviors, such as healthier eating habits, smoking cessation, or choosing science, technology, engineering, and math (STEM) career paths. In all of these contexts, a narrative message being an oversimplification and engaging a narrative way of thinking doesn’t much matter if the larger goal is met: was knowledge learned? Were attitudes or behaviors changed?

Such goal-directed uses of narrative messages, of course, come with caveats. There might be societal disagreement about what kinds of behaviors or attitudes should be encouraged, raising ethical questions about what kinds of advocacy by scientists are appropriate [9,10]. Engaging in a battle over competing narrative messages in larger public debates pits scientific communication against narrative messages constructed by other actors. Competing narrative messages potentially introduce counter arguments or even misinformation, while competing at the same level with narrative messages constructed around the best scientific evidence.

### Scientific reasoning

If the goal of communicating science is instead to help an audience engage or develop scientific reasoning and orient discourse around evidence, narrative messages may be counterproductive. In this context, a narrative message being an oversimplification matters greatly because, by engaging a narrative way of thinking, the communication fails to reach the larger goal of engaging scientific reasoning. Since narrative messages can be persuasive regardless of the validity of underlying factual claims, the use of narrative messages in this context becomes an oversimplification at best or manipulative at worst. Even an otherwise desired outcome (such as correct knowledge about climate change) could be viewed negatively if the desired reasoning process was not engaged.

If scientific reasoning is developed or activated in an audience, other communicators within public debates would find it more difficult to compete through narrative messages because the “manipulation" might become more apparent to audiences. Competing communicators might instead try to use scientific reasoning to counter, which scientists in this context would likely view as valid rebuttals.

Hence, the paradox comes into focus—storytelling can meaningfully engage audiences and make scientific information relevant while simultaneously encouraging a narrative way of thinking that places scientific stories on a similar level to any other plausible story that may or may not support scientific truth. Abandoning storytelling for the sake of scientific reasoning merely abdicates the influence of a powerful communication technique to nonscience communicators. Yet storytelling also fails to engage the way of evaluating evidence that has given science its rightful claim to truth.

## Escaping the paradox?

While our discussion thus far has treated a narrative way of thinking as often being at odds with scientific reasoning, it is possible to use the first in service of the second. Narrative messages can be constructed toward the goal of engaging audiences to understand the process and credibility of scientific reasoning. These types of narratives might tell the story of a scientist who uses scientific reasoning to arrive at an important result. They might tell the story of a character who struggles to overcome a conflict until scientific reasoning is used. They show the process of science through an individual’s experience.

This is not a new idea, as talented science journalists often make this connection in their work. Nor is this a panacea to the paradox. Increased scientific literacy does not lead to greater acceptance of science [11,12], so merely knowing more about the scientific method is unlikely to solve the problem on its own. Yet we think this middle path offers one way to deconstruct the paradox to see how science can use storytelling while still emphasizing its right to truth claims. In the end, using storytelling to primarily build scientific support through knowledge, attitude, or behavior goals without also engaging scientific reasoning might not help science in the long run.

The latest National Science Foundation (NSF) Science and Engineering Indicators, a biennial report measuring the state of science in the United States, finds only one in four Americans (26%) are able to correctly describe what constitutes a scientific study. Surveys from China show similarly low numbers with less than a third (31%) of respondents understanding the idea of scientific research [13]. A limited understanding of the scientific process is particularly troubling in light of ongoing discussions about replicability and reproducibility in psychology and other scientific fields [14–16] in which perceptions can cast doubt on the enterprise of science itself rather than promote discussion about how science can best use new computational tools and methods to improve on its self-correcting nature.

Unless the scientific community also focuses on these long-term narratives to help build understanding of the process of scientific knowledge production, the facts or conclusions it puts forth might increasingly be seen on equal footing with arguments and judgments offered by other societal stakeholders [17]. This is particularly worrisome in a world where the idea that the scientific process is the best way of producing valid and reliable information is increasingly under siege. For science, narratives might have most of their power not in conveying facts or building excitement but in rebuilding the foundation of understanding scientific reasoning.

Societal buy-in to science’s unique claim to producing the best available evidence depends on citizens appreciating the process that underlies science. By using narrative more narrowly to fight for small victories over science knowledge instead of using narrative to build understanding of the process of scientific reasoning, science may remain caught up in this paradox and risk its place as our societal curator of knowledge.

## Abbreviations

NSF: National Science Foundation
STEM: science, technology, engineering, and math
